# A new species of the genus *Sulawesifulvius* Gorczyca, Chérot, & Štys, 2004 (Insecta, Heteroptera, Miridae, Cylapinae) from India

**DOI:** 10.3897/zookeys.475.8349

**Published:** 2015-01-22

**Authors:** H. M. Yeshwanth, Chérot Frédéric

**Affiliations:** 1 Department of Agricultural Entomology, University of Agricultural Sciences, GKVK, Bangalore 560 065, India; 2 Département de l’Etude du Milieu Naturel et Agricole, Service Public de Wallonie, Gembloux, BE-5030, Belgium

**Keywords:** *Sulawesifulvius indicus* sp. n., Cylapinae, India, taxonomy

## Abstract

A new species, *Sulawesifulvius indicus* **sp. n.**, is described from Bangalore, India. It is easily separated from the type species *Sulawesifulvius schuhi* Gorczyca et al., 2004, the only other species of the genus, by the small size, dorsal coloration, and the male genital structures. The discovery of a new species of *Sulawesifulvius* in southern India considerably extends the distribution of the genus, previously recorded only from Sulawesi, Indonesia.

## Introduction

The monotypic genus *Sulawesifulvius* was erected by Gorczyca et al. (2004) to accommodate the new plant bug species *Sulawesifulvius schuhi* Gorczyca, Chérot & Štys, 2004 from Nani Wartabone National Park, Sulawesi (Indonesia).

Recently, two specimens of an unknown cylapine were collected by the first author in Hessaraghatta, near Bangalore, South India. These Indian specimens conform to the original generic diagnosis of the genus *Sulawesifulvius*. However, the genital structures of the male holotype, especially the parameres, are different and much simpler than the male genitalia of *Sulawesifulvius schuhi*. Consequently, we describe these specimens as a new species of *Sulawesifulvius*.

## Material and methods

The specimens examined for the study are deposited in the collection of the Department of Entomology, University of Agricultural Sciences, Bangalore, India (UASB). The terminology adopted for male genitalia follows Gorczyca et al. (2004).

All measurements are given in millimeters. Photographs were taken using a Leica M205 C microscope. Multiple images were taken at different depths and were combined using a Combine ZM software. Illustrations of male genitalia were drawn using a Leica DM2000 compound microscope attached to a camera lucida.

## Results

### Sulawesifulvius indicus sp. n.

Taxon classificationAnimaliaHemipteraMiridae

http://zoobank.org/4D0C4992-1485-4F85-BDB0-3AE32B840CE6

Figs 1–4 , 5–7 , 8–9

#### Type material.

Holotype ♂: India: Karnataka: Hessarghatta, near Bangalore (approx. 13°09'N, 77°29'E; altitude: 960 m), 20.vi.2011, at light, H.M. Yeshwanth leg. (UASB). Paratype ♀: India: Karnataka, Hessaraghatta, near Bangalore, 2.v.2010, at light, H.M. Yeshwanth leg. (UASB).

#### Diagnosis.

**Figures 1–4.**
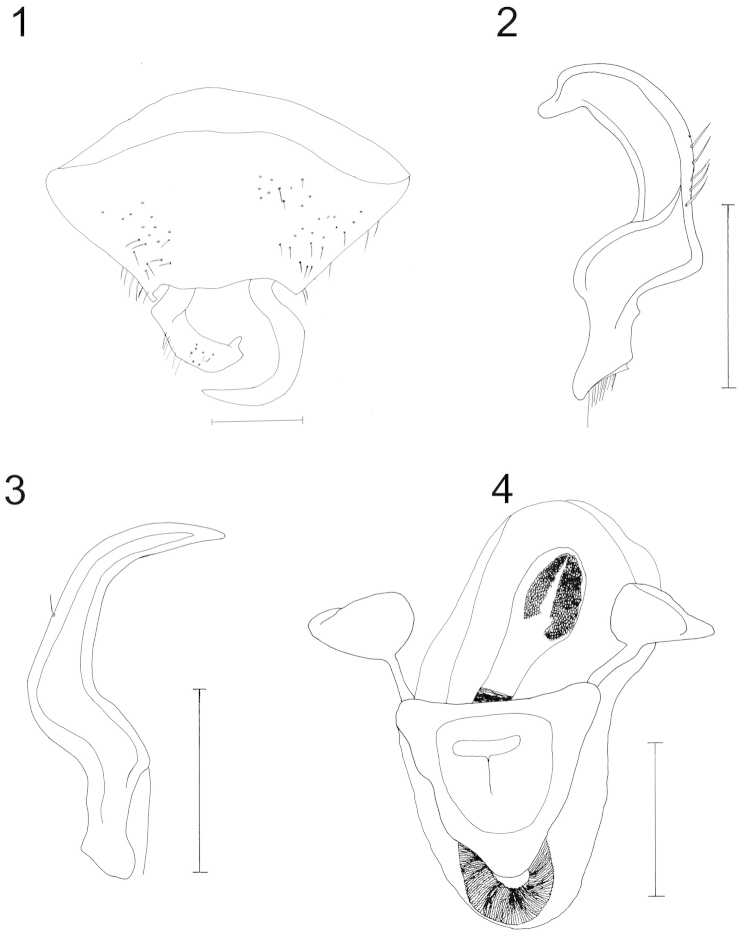
*Sulawesifulvius indicus* sp. n. male holotype, genital structures. **1** pygophore (dorsal view). Scale = 0.5 mm. **2** left paramere **3** right paramere **4** phallus Scales = 0.25 mm.

Small species, length 2.70, width 1.50 (versus length 3.40 and width 1.80 in *Sulawesifulvius schuhi*), dorsally yellowish brown, slightly tinged with dark red, with simple parameres (Figs 2–3), devoid of sharp processes (versus parameres with sharp processes in *Sulawesifulvius schuhi*), and pygophore with a straight right side and a posterior margin not deeply curved (Fig. 1).

#### Description.

***Body of male*** pale yellow with pale red and brown markings; body length 2.70, width 1.50 (Fig. 9).

***Head*** pale yellow, longer than width, clypeus prominent; vertex and frons with two pairs of tubercles; head length in dorsal view 0.40, intraocular width 0.27; first segment of antenna tubular, pale yellow, tinged with red and brown markings; second segment yellow with a brown band medially, covered with short, pale setae; third segment pale yellow, apex dark brown, with bright white setae; fourth segment dark brown, with bright white setae; length of antennal segments: 0.15: 0.30: 0.40: 0.15; rostrum brown, shiny, length of rostral segments: 0.22: 0.25: 0.22: 0.25.

***Pronotum*** pale yellow with red and brown markings, anterior margin of pronotum concave, with two brown spots; calli raised, large, tuberculate; lateral margins with brown spots on posterior region; anterolateral angle with scalelike setae; posterior margin arcuate. Length of pronotum 0.39, width of pronotum 1.18, length along lateral margin 0.50.

**Figures 5–7.**
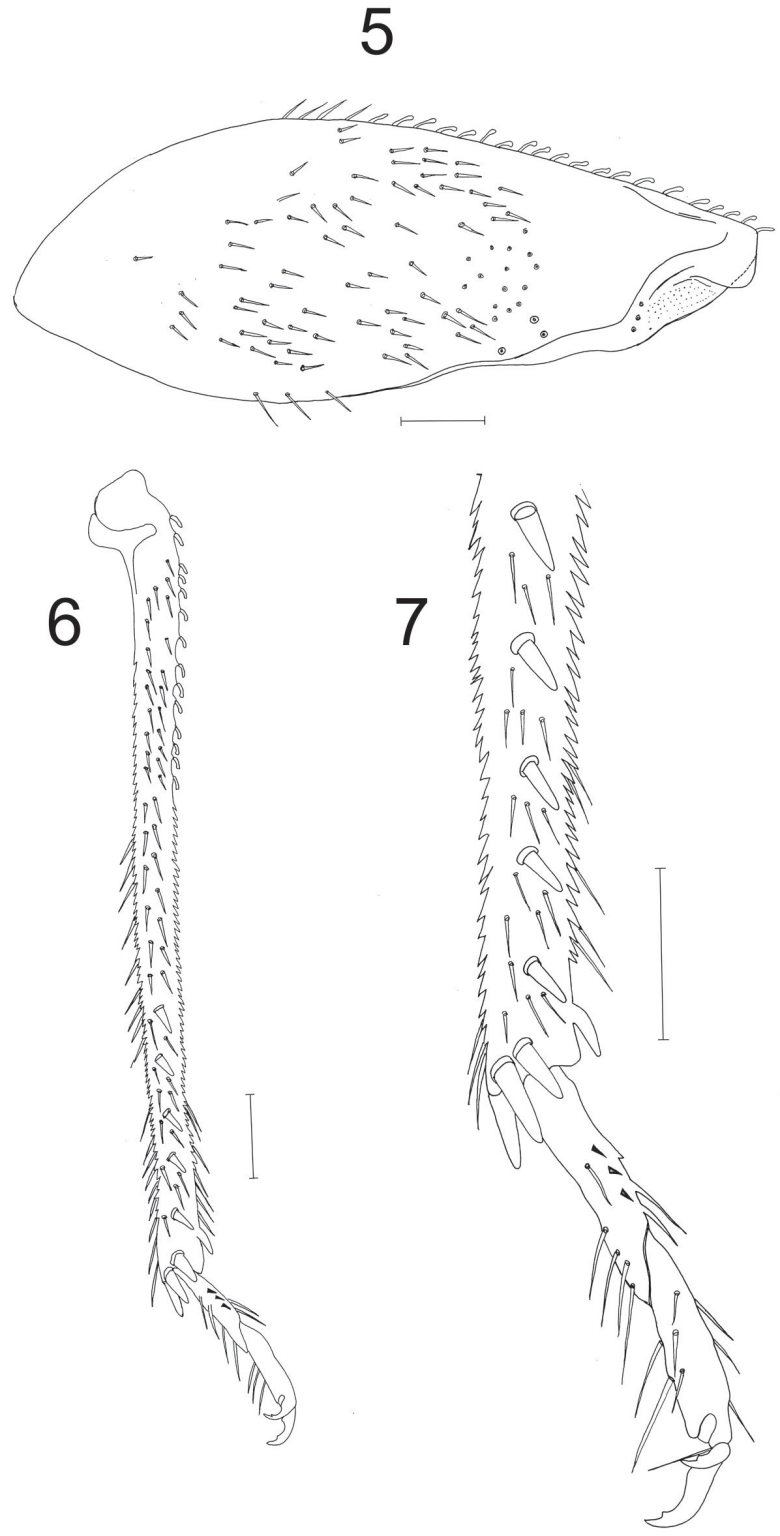
*Sulawesifulvius indicus* sp. n. male holotype, legs. **5** metafemur **6** metatibia and tarsus. Scales = 0.5 mm **7** apex of metatibia and tarsus. Scale = 0.25 mm.

***Legs*** (Figs 5–7) coxae, trochanters and femora pale yellow, their apices with red patches; femora swollen; metafemur greatly enlarged with a transverse reddish band and three trichobothria; metatibia with scalelike and long setae and longitudinal rows of short spines on each side and thick spines on apical region; tarsus two segmented; metatarsus with short, thick spines; parempodia setiform; claw with subapical tooth.

***Mesoscutum*** exposed, yellowish brown, with brown patches and tinged with red on sides.

***Scutellum*** pale yellow, with a longitudinal pale stripe medially; apex with red markings.

***Hemelytra*** pale yellow, with short shining scalelike setae; corium with brown pattern reaching cuneal incisure, outer margin of embolium with brown patches; cuneus broadly triangular, with brown patches reaching apex of membrane; membrane whitish, tinged with brown.

***Ventral surface*** pale yellow, with red bands.

***Pygophore*** basally broad and narrow toward apex (Fig. 1).

***Parameres*** simple, left paramere flat, strongly curved, with a basal sensory lobe, apex rounded, beaklike (Fig. 2); right paramere simple, slightly larger than left paramere, C-shaped with an apex gradually narrowing distally (Fig. 3).

***Phallus*** prominent (Fig. 4); *ductus seminis* not sclerotized, flexible with terminal circular sclerotized opening; theca membranous, covering endosoma, apex of endosoma globular, with numerous small sclerotized, interconnected processes or rounded structures.

**Figures 8–9.**
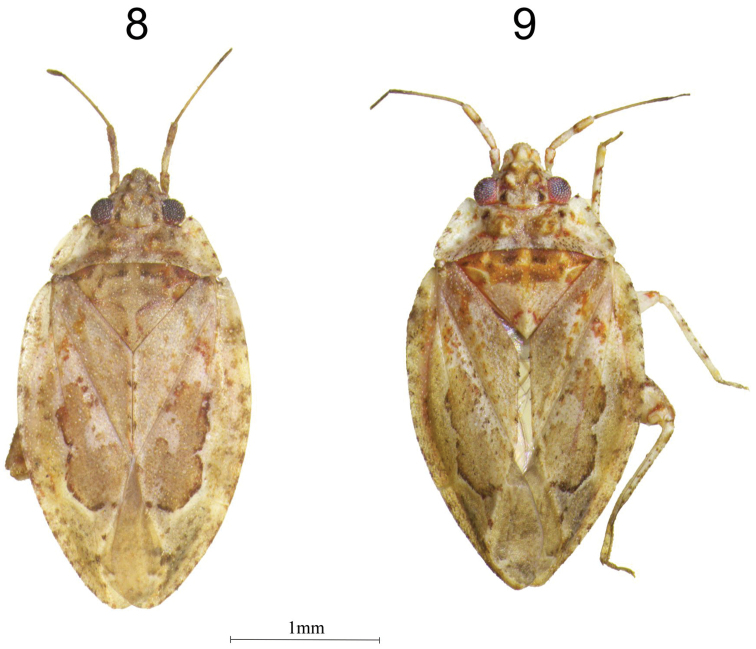
*Sulawesifulvius indicus* sp. n. habitus in dorsal view. **8** female paratype **9** male holotype.

***Body of female*** very similar to that of male in shape, size, color, and vestiture (Fig. 8).

#### Etymology.

The name of the species refers to India, the country where it was collected (adjective derived from the geographical name).

#### Habitat.

The habitat surrounding street lamps consisted of trees dominated by tamarind (*Tamarindus indica* Linné) (Caesalpiniaceae), few trees of neem (*Azadirachta indica* A. Juss) (Meliaceae), *Acacia* sp., and bushes dominated by lantana (*Lantana camera* Linné) (Verbenaceae).

## Discussion

*Sulawesifulvius indicus* sp. n. is similar to *Sulawesifulvius schuhi*, the type species and only other species known for the genus (Gorczyca 2006a, Schuh 2002–2013 online). Both species are similar and could be confused on the basis of their external anatomy alone, in particular the body shape (small, oval, flattened dorsoventrally), the head structure (relatively short, inserted in anterior part of pronotum), the vertex with two raised tubercles, the prominent clypeus in dorsal view, the antennal shape (first antennal segment thick, shorter than the head, second antennal segment slightly narrowed in the middle, third antennal segment the longest and thinnest, fourth antennal segment spindle shaped), the pronotal shape and structure (lateral margins elevated, anterior margin enveloping eyes posteriorly, calli wide, raised, totally separated), the metafemoral shape (broad, with subapical depression on side) and the hemelytral structure (exocorium broad, cuneus elongate, partially enveloping membrane, characteristic pattern and vestiture). These species differ by the smaller size of *Sulawesifulvius indicus* sp. n., the differences in coloration (their pattern are very similar but the dorsal coloration is yellowish brown, slightly tinged with red, in *Sulawesifulvius indicus*; yellowish on the hemelytra and reddish on the head, pronotum and scutellum in *Sulawesifulvius sulawesicus*), the male genital structures, particularly by the parameres (simple in *Sulawesifulvius indicus*, with sharp processes in *Sulawesifulvius schuhi*), and the shape of the pygophore.

The discovery of a new species of *Sulawesifulvius* in southern India considerably extends the distribution of the genus, previously recorded only from Sulawesi, Indonesia but does not modify significantly the original diagnosis of the genus (Gorczyca et al. 2004).

As noted by Gorczyca et al. (2004) a remote, superficial similarity can be observed between the genera *Peritropis* Uhler, 1891 and *Sulawesifulvius*. However, these genera can be easily separated by the structure of head, antennae, pronotum, legs, and hemelytra. Recent revisions of *Peritropis* for the Old (Gorczyca 2000, 2006b) and New Worlds (Wolski and Henry 2012) confirm this conclusion.

## Supplementary Material

XML Treatment for Sulawesifulvius indicus
